# c-KIT receptor expression is strictly associated with the biological behaviour of thyroid nodules

**DOI:** 10.1186/1479-5876-10-7

**Published:** 2012-01-10

**Authors:** Sara Tomei, Chiara Mazzanti, Ivo Marchetti, Leonardo Rossi, Katia Zavaglia, Francesca Lessi, Alessandro Apollo, Paolo Aretini, Giancarlo Di Coscio, Generoso Bevilacqua

**Affiliations:** 1 Section of Molecular Pathology, Division of Surgical, Molecular, and Ultrastructural Pathology, University of Pisa and Pisa University Hospital, via Roma 57, Pisa, 56100, Italy; 2 Section of Cytopathology, Division of Surgical, Molecular, and Ultrastructural Pathology, University of Pisa and Pisa University Hospital, via Roma 57, Pisa, 56100, Italy; 3 Department of Human Morphology and Applied Biology, University of Pisa and Pisa University Hospital, via Volta 4, Pisa, 56100, Italy

**Keywords:** c-KIT, thyroid cancer, FNAC

## Abstract

**Background:**

A large amount of information has been collected on the molecular tumorigenesis of thyroid cancer. A low expression of c-KIT gene has been reported during the transformation of normal thyroid epithelium to papillary carcinoma suggesting a possible role of the gene in the differentiation of thyroid tissue rather than in the proliferation. The initial presentation of thyroid carcinoma is through a nodule and the best way nowadays to evaluate it is by fine-needle aspiration (FNA). However many thyroid FNAs are not definitively benign or malignant, yielding an indeterminate or suspicious diagnosis which ranges from 10 to 25% of FNAs. BRAF mutational analysis is commonly used to assess the malignancy of thyroid nodules but unfortunately it still leaves indeterminate diagnoses. The development of molecular initial diagnostic tests for evaluating a thyroid nodule is needed in order to define optimal surgical approach for patients with uncertain diagnosis pre- and intra-operatively.

**Methods:**

In this study we extracted RNA from 82 FNA smears, 46 malignant and 36 benign at the histology, in order to evaluate by quantitative Real Time PCR the expression levels of c-KIT gene.

**Results:**

We have found a highly preferential decrease rather than increase in transcript of c-KIT in malignant thyroid lesions compared to the benign ones. To explore the diagnostic utility of c-KIT expression in thyroid nodules, its expression values were divided in four arbitrarily defined classes, with class I characterized by the complete silencing of the gene. Class I and IV represented the two most informative groups, with 100% of the samples found malignant or benign respectively. The molecular analysis was proven by ROC (receiver operating characteristic) analysis to be highly specific and sensitive improving the cytological diagnostic accuracy of 15%.

**Conclusion:**

We propose the use of BRAF test (after uncertain cytological diagnosis) to assess the malignancy of thyroid nodules at first, then the use of the c-KIT expression to ultimately assess the diagnosis of the nodules that otherwise would remain suspicious. The c-KIT expression-based classification is highly accurate and may provide a tool to overcome the difficulties in today's preoperative diagnosis of thyroid suspicious malignancies.

## Introduction

Thyroid carcinoma represents 90% of all endocrine malignancies and about 1% of all human malignancies, with an increasing incidence [1]. Papillary thyroid carcinoma (PTC) is its most frequent histotype and usually its first clinical presentation is a thyroid nodule [2,3]. Five to 10% of the population will develop a clinically significant thyroid nodule during their lifetime [4], whereas PTC accounts only for 5% of all thyroid nodules [5].

A large amount of information has been accumulated on the molecular pathogenesis of thyroid cancer [6]. For what concerns PTC, the activation of MAPK signalling pathway is a major event, as a result either of BRAF or RAS point mutations or RET/PTC rearrangement.

The proto-oncogene c-KIT is a type III receptor tyrosine-kinase, cellular homolog of the viral oncogene of the feline sarcoma retrovirus HZ4-FeSV. It plays various roles in haematopoiesis, melanogenesis and spermatogenesis, and in the development of the interstitial cells of Cajal. Its ligand is the stem cell factor (SCF) [7,8]. The role of c-KIT in human neoplasia is not fully cleared yet. A number of tumour types are associated with activation of c-KIT through its overexpression or through activating mutations [7,9-11], while in highly metastatic melanomas, breast cancer and thyroid carcinoma the progression into a malignant phenotype correlates mostly with loss of c-KIT expression [12-16]. Among the few papers studying c-KIT status in thyroid cancer, Natali *et al*. in 1995 [17] reported the loss of the receptor during the transformation of normal thyroid epithelium to papillary carcinoma. Similarly, in 2004 Mazzanti *et al*. [18], by using a microarray assay, were able to identify, out of thousands of genes, c-KIT as one of the most significant down-expressed gene in PTC compared to benign lesions. Other laboratories confirmed this result by using quantitative Real-Time PCR (qPCR) [19,20]. Moreover, multiple miRNAs, predicted to target c-KIT, have been reported to be up-regulated in PTC [21,22]. These findings indicate that the c-KIT receptor may be involved in the growth control of thyroid epithelium and that this function may be lost in malignant transformation.

Nowadays, thyroid fine-needle aspiration cytology (FNAC) is the best available test in the evaluation of a thyroid nodule [23,24] and has greatly reduced the need for thyroid surgery. Unfortunately, a percentage of FNAC (~ 30%) is suspicious for papillary carcinoma (SPTC) or reveals an indeterminate follicular proliferation (IFP), not allowing a sure diagnosis of malignity or benignity [25-28]. According to the Guidelines for the Management of Thyroid Cancer, British Thyroid Association, SPCT corresponds to the abbreviation Thy4 and IFP to Thy3.

As in other diseases, molecular pathology is playing a relevant role in diagnosis of thyroid cancer. Recently, several studies have demonstrated that the BRAF V600E mutation represents a diagnostic and prognostic biomarker in PTC, with a prevalence of 40-66%, whereas it is never found in benign lesion [29,30]. Very recent papers of our laboratory have proposed a new simple method, named manual macrodissection, to perform molecular analysis on cells obtained by FNAC, and have demonstrated the usefulness of the association cytology-molecular biology for PTC and micro-PTC diagnosis. In particular, the analysis of the BRAF gene status increases the diagnostic accuracy for PTC of 20-30%. However BRAF V600E analysis still leaves a percentage of SPTC and IFP, urging the finding of other molecular markers.

The present study evaluates c-KIT expression in a morpho-molecular diagnostic approach to a series of thyroid FNAC, together with the study of BRAF gene status. We confirm the down regulation of c-KIT in PTC, and show that c-KIT analysis can have a diagnostic role in thyroid FNAC.

## Materials and methods

### Specimens

Preoperative FNAC slides of 82 thyroid nodules from as many patients were selected from files of the Section of Cytopathology, Division of Surgical, Molecular and Ultrastructural Pathology, University of Pisa, collected from 2003 to 2010. For ethical reasons, only cases with extra slides were used. Selected cells (neoplastic, SPTC or IFP) were manually scraped with a method previously described, named manual macrodissection [29].

### Ethical Board

This study was approved by the Internal Review Board of the University of Pisa. All patients gave their consent for the participation to the study.

### Diagnosis

Cytological diagnosis was based on the following criteria, broadly suggested in the literature: smear background, cell arrangements, cell shape, nuclear/cytoplasmic features, presence of nucleoli and mitosis. In particular, the cytological diagnosis of IFP was based on the presence of microfollicular pattern, irregularity of structure and absence of colloid, whereas nuclear atypia and mitosis were not present. Histological diagnosis assessed ultimately the malignity or benignity of all lesions.

Smears were independently reviewed by senior cytopathologists to assure adequate thyroid cell representation of the slides in which molecular analysis was performed.

The histological diagnosis of the 82 samples collected was of PTC in 46 cases and of benign nodule in 36 cases. As described in Table 1, of the 46 cases with a definitive histological diagnosis of PTC, the cytological diagnosis was of PTC in 30 cases (65%), of SPTC in 11 cases (24%) and of IFP in 5 cases (11%). In the 36 cases with a definitive histological diagnosis of benign nodule, the cytological diagnosis was of benign nodule (BN) in 17 cases (47%) and of IFP in 19 cases (53%).

**Table 1:** Histological and cytological diagnoses of 82 thyroid nodules

*Histological Diagnosis*	*Cytological Diagnosis*		
	**PTC**	**SPTC**	**IFP**
	
**PTC: 46 cases**	**30 (65%)**	**11 (24%)**	**5 (11%)**

	**BN**	**IFP**	
		
**BN: 36 cases**	**17 (47%)**	**19 (53%)**	

PTC: papillary thyroid carcinoma. SPTC: suspicious for PTC. IFP: indeterminate follicular proliferation. BN: benign nodule.

### DNA and RNA extraction

Archival FNAC slides stained with Papanicolaou technique were kept in xylene for 1 to 3 days, depending on the time of storage, in order to detach the slides coverslips. Slides were then hydrated in a graded series of ethanol baths, followed by a wash in distilled H_2_O for 1 minute and finally air-dried. DNA extraction was performed using a commercially available kit (Nucleospin, Macherey-Nagel, Düren, Germany) with a modification to the first step. Fifty percent of the lysis solution without proteinase K was initially poured on the slide to scrape off the cytological stained sample using a single edged razor blade. Any scraped tissue was then collected in a microcentrifuge tube containing the other half of the lysis solution with Proteinase K. The extracted DNA was kept at -20° until used. RNA extraction was performed by using a commercial kit (High Pure RNA Paraffin kit, Roche). The quantity/quality of extracted RNA and DNA was estimated with Nanodrop 1000 spectrophotometer by using 1 μl of undiluted RNA/DNA solution. RNA was then reverse transcribed in cDNA in a final volume of 20 μl, containing 5X RT buffer, 10 mM dNTPs, 50 ng/μl Random Primers, 0.1 M DTT, 40 U/μl RNaseOUT, 50 μM oligo(dT), DEPC-Treated Water, 15 U/μl Cloned AMV reverse transcriptase (Invitrogen, Carlsbad, CA).

### c-KIT mRNA expression analysis

The level of c-KIT expression was analysed by quantitative Real-Time PCR (qPCR) on the Rotor-Gene 6000 real time rotary analyzer (Corbett, Life Science, Australia) following the manufacturing instructions. Endogenous reference gene (B2M, beta 2 microglobulin) was used to normalize each gene expression level. PCR was performed in 25 μl final volume, containing 5 μl of cDNA, 12.5 μl of MESA GREEN qPCR MasterMix Plus (EUROGENTEC, San Diego, CA), 300 nM of each primer (Invitrogen, Carlsbad, CA) with the following cycling conditions: initial denaturation 95°C for 5 min; 40 cycles at 95°C for 15 sec and 58°C for 40 sec and 72°C for 40 sec; final step 25°C for 1 min. Primers for c-KIT and B2M amplification were selected using Primer3 software:

- c-KIT F: 5'- GCACCTGCTGCTGAAATGTATGACATAAT-3'

- c-KIT R: 5'- TTTGCTAAGTTGGAGTAAATATGATTGG-3'

- B2M F: 5'- CATTCCTGAAGCTGACAGCATTC-3'

- B2M R: 5'- TGCTGGATGACGTGAGTAAACC-3'

A first PCR run was performed on control c-KIT expressing sample and run on 2% agarose gel. The PCR product was excised from the gel, purified by using GenElute™ PCR Clean-Up (Sigma-Aldrich) and measured spectrophotometrically at 260 and 280 nm.

The purified product was diluted in a 10-fold series to create the standards for a ten-point standard curve that was run in triplicate. Standard curves were generated for both c-KIT and B2M and showed a good linearity with consistent correlation coefficient (R^2 ^= 0.999). Ct was determined by the Rotor-Gene 6000 software and exported for analysis after background subtraction. Threshold was set by standard curve and then imported in all the runs for data analysis. PCR efficiencies resulted similar for both c-KIT and B2M in each experiment and ranged between 98-102%. The experiment was run in duplicate for each sample.

To verify primers specificities, melting curve analysis was performed. Fluorescent data were acquired during the extension phase. After 40 cycles a melting curve for each gene was generated by slowly increasing (0.1°C/s) the temperature from 60°C to 95°C, while the fluorescence was measured. For each experiment a no-template reaction was included as a negative control.

c-KIT expression was ultimately represented as the ratio of absolute quantification by standard curve of c-KIT expression and B2M expression.

### c-KIT genotyping

c-KIT sequence was screened for mutations in exons 9, 11, 13 and 17 by direct sequencing. PCR was performed using standard conditions: initial denaturation 95°C for 7 min; 40 cycles at 95°C for 45 sec and 56°C for 45 sec and 72°C for 45 sec; final step 72°C for 10 min with AmpliTaq Gold (Applied Biosystems) on 9700 GeneAmp PCR System (Applied Biosystems). Primers for c-KIT sequencing were selected using Primer3 software:

- exon 9 F: 5'- CCAGGGCTTTTGTTTTCTTC-3'

- exon 9 R: 5'- TGGTAGACAGAGCCTAAACATCC-3'

- exon 11 F: 5'- GATCTATTTTTCCCTTTCTC-3'

- exon 11 R: 5'- AGCCCCTGTTTCATACTGAC-3'

- exon 13 F: 5'- TCAGTTTGCCAGTTGTGCTT-3'

- exon 13 R: 5'- AATGTCATGTTTTGATAACCT-3'

- exon 17 F: 5'- TTCTTTTCTCCTCCAACCTAA-3'

- exon 17 R: 5'- TGTCAAGCAGAGAATGGGTA-3'

The PCR products were purified with Multi Screen PCR Plates (Millipore) and the sequencing reactions were performed in 20 μl final volume using Big Dye Terminator kit v3.1 (Applied Biosystems) and 2.5 pmol/μl of each primer, then purified with Multi Screen PCR Plates (Millipore). The sequence reactions were loaded on ABI PRISM 3100 Genetic Analyzer (Applied Biosystems) and analyzed using the Sequencing Analysis software 3.4 version.

### BRAF V600E mutational status

The *BRAF *V600E mutational status was determined by automated pyrosequencing analysis (Qiagen).

### Statistical analysis

Student's t-test was used to compare the mean of c-KIT expression between malignant and benign samples. Chi-square test was used to statistically analyse each single class of c-KIT versus the sum of the other ones. Then malignity and benignity indexes, which represent the percentage of cases with a malignant or benign diagnosis in each class, were calculated and the relative p-value was reported. To better estimate the significance of the malignity index trend we also performed a logistic regression. All the analyses were performed by using Statgraphics Centurion (V. 15, StatPoint, Inc.). In order to evaluate the Area Under the Curve (AUC) as a measure of sensitivity and specificity of our method, we used a ROC (receiver operating characteristic) curve analysis (Medcalc 11, Medcalc Software). To evaluate the statistical significance of the change in diagnostic accuracy before and after performing molecular analyses we used Chi-square test for comparison of two proportions (independent samples) expressed as a percentage.

## Results

### Expression and genotyping of c-KIT receptor in benign and malignant thyroid lesions

c-KIT expression was analyzed by qPCR in a set of 82 FNAC, histologically diagnosed as 36 benign and 46 malignant thyroid nodules (Table 2). Overall, c-KIT expression was detected in 59% of PTC (27/46) and in 100% of BN (36/36). The mean of c-KIT expression values (c-KIT/B2M ratio) was calculated for both benign (1.72) and malignant (0.138) groups and the difference resulted highly significant (p < 0.0001).

**Table 2:** Morphological and molecular diagnosis in 82 thyroid nodules

case	HD	CD	c-KIT Class	c-KIT ev	BRAF
1	PTC	PTC	I	0	V600E

2	PTC	PTC	I	0	V600E

3	PTC	PTC	I	0	V600E

4	PTC	PTC	I	0	V600E

5	PTC	PTC	I	0	V600E

6	PTC	PTC	I	0	V600E

7	PTC	PTC	I	0	V600E

8	PTC	PTC	I	0	V600E

9	PTC	PTC	I	0	V600E

10	PTC	PTC	I	0	V600E

11	PTC	PTC	I	0	V600E

12	PTC	PTC	I	0	V600E

13	PTC	PTC	I	0	WT

14	PTC	PTC	I	0	WT

15	PTC	PTC	I	0	WT

16	PTC	PTC	II	0.5	V600E

17	PTC	PTC	II	0.448	V600E

18	PTC	PTC	II	0.105	V600E

19	PTC	PTC	II	0.07	V600E

20	PTC	PTC	II	0.0533	V600E

21	PTC	PTC	II	0.049	V600E

22	PTC	PTC	II	0.031	V600E

23	PTC	PTC	II	0.022	WT

24	PTC	PTC	II	0.013	V600E

25	PTC	PTC	II	0.0126	WT

26	PTC	PTC	II	0.01	WT

27	PTC	PTC	II	0.004	WT

28	PTC	PTC	II	0.0003	WT

29	PTC	PTC	II	0.0002	V600E

30	PTC	PTC	III	1.0506	WT

31	PTC	SPTC	I	0	WT

32	PTC	SPTC	I	0	WT

33	PTC	SPTC	I	0	WT

34	PTC	SPTC	I	0	WT

35	PTC	SPTC	II	0.44	V600E

36	PTC	SPTC	II	0.42	V600E

37	PTC	SPTC	II	0.022	V600E

38	PTC	SPTC	II	0.011	V600E

39	PTC	SPTC	II	0.42	WT

40	PTC	SPTC	II	0.37	WT

41	PTC	SPTC	III	1.27	WT

42	PTC	IFP	II	0.275	WT

43	PTC	IFP	II	0.097	WT

44	PTC	IFP	II	0.07	WT

45	PTC	IFP	II	0.023	WT

46	PTC	IFP	III	0.57	WT

47	BN	BN	II	0.3	WT

48	BN	BN	II	0.14	WT

49	BN	BN	II	0.07	WT

50	BN	BN	II	0.1079	WT

51	BN	BN	II	0.363	WT

52	BN	BN	III	0.561	WT

53	BN	BN	III	0.6457	WT

54	BN	BN	III	0.858	WT

55	BN	BN	III	0.96	WT

56	BN	BN	III	0.97	WT

57	BN	BN	III	1.051	WT

58	BN	BN	III	1.3823	WT

59	BN	BN	III	1.39	WT

60	BN	BN	III	2.47	WT

61	BN	BN	III	2.5083	WT

62	BN	BN	III	2.6333	WT

63	BN	BN	IV	7.24	WT

64	BN	IFP	II	0.032	WT

65	BN	IFP	II	0.04	WT

66	BN	IFP	II	0.0463	WT

67	BN	IFP	II	0.0599	WT

68	BN	IFP	II	0.1143	WT

69	BN	IFP	II	0.1957	WT

70	BN	IFP	II	0.217	WT

71	BN	IFP	II	0.2619	WT

72	BN	IFP	III	0.7302	WT

73	BN	IFP	III	0.84	WT

74	BN	IFP	III	0.948	WT

75	BN	IFP	III	1.09	WT

76	BN	IFP	III	1.3727	WT

77	BN	IFP	III	1.64	WT

78	BN	IFP	III	1.67	WT

79	BN	IFP	IV	3.4	WT

80	BN	IFP	IV	7.69	WT

81	BN	IFP	IV	8.73	WT

82	BN	IFP	IV	9.34	WT

HD: histological diagnosis. CD: cytological diagnosis. c-KIT ev: c-KIT expression value. PTC: papillary thyroid carcinoma. SPTC: suspicious for PTC. BN: benign nodule. IFP: indeterminate follicular proliferation. WT: wild type.

The sequencing of exons 9, 11, 13, 17 of the c-KIT resulted wild-type for all the samples analyzed.

### c-KIT expression and biological behavior of thyroid nodules

The value of c-KIT expression (c-KIT/B2M ratio) ranged between 0 and 9.34 (Table 2). To evaluate a possible relationship with the biological behavior of lesions, c-KIT expression values (ev) were arbitrarily organized in four classes (Table 3):

**Table 3:** Classes of c-KIT expression value

Class	c-KIT ev	PTC		BN		p value
			
		n	%	n	%	
I	0	19	100	0	0	< 0.0001

II	> 0-≤ 0.5	24	65	13	35	= 0.1400

III	> 0.5-≤ 3.0	3	14	18	86	< 0.0001

IV	> 3	0	0	5	100	= 0.0091

		46		36		

c-KIT ev: c-KIT expression value. PTC: papillary thyroid carcinoma. BN: benign nodule.

- Class I: c-KIT ev = 0;

- Class II: c-KIT ev > 0 and ≤ 0.5;

- Class III: c-KIT ev > 0.5 and ≤ 3;

- Class IV: c-KIT ev > 3;

The percentage of malignant and of benign cases was calculated in each class and its statistical significance was determined (p-value).

In class I the percentage of malignancy is 100% (19/19 cases), whereas in class IV the percentage of benignity is 100% (5/5). In class II the percentage of malignant cases is higher than benign cases: 65% (24/37) vs 35% (13/37). On the other hand, class III has a higher percentage of benign cases than malignant ones: 86% (18/21) vs 14% (3/21). Difference between malignant and benign lesions is statistically highly significant in classes I, IV and III (p < 0.0001, p = 0.0091, p < 0.0001). p-value in class II is 0.14.

**Figure 1:**
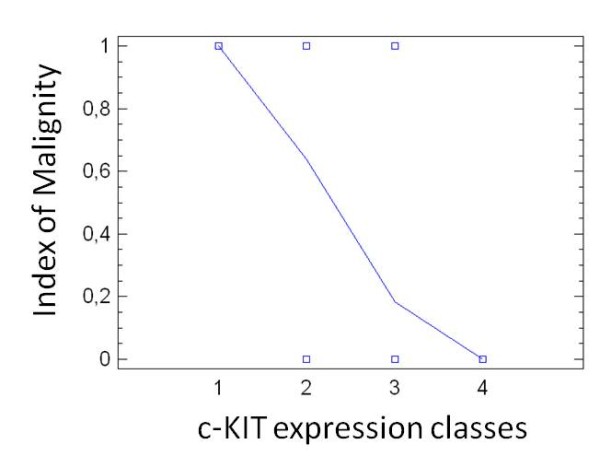
**Logistic regression**. Logistic regression model describing the relationship between the risk of malignancy and the classes of c-KIT expression. The p-value of the model resulted to be less than 0.05.

**Figure 2:**
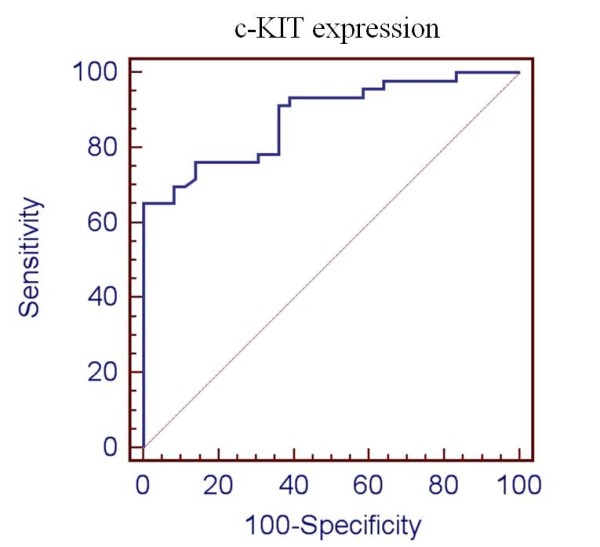
**ROC analysis for c-KIT expression**. The true positive rate (Sensitivity) is plotted in function of the false positive rate (100-Specificity) for different cut-off points. Each point on the ROC plot represents a sensitivity/specificity pair corresponding to a particular decision threshold. (AUC = 0.9, C.I 95% 0.79-.94; p = 0.001).

Figure 1 reports the results of fitting a logistic regression model. The p-value of the diagnostic model is less than 0.05, showing a statistically significant relationship between the variables at the 95% confidence level. Specificity and sensitivity of the diagnostic performance of the model were evaluated by ROC analysis and the AUC was 0.881, with C.I. 95% 0.79-0.94 and p = 0.001, indicating that the model has a statistically significant efficacy in discriminating malignant from benign lesions (Figure 2).

### c-KIT expression in FNAC

The cytological diagnoses of the 46 histologically confirmed PTCs were distributed through the 4 classes as following (Table 4):

**Table 4:** Distribution of cytological diagnosis in the c-KIT expression classes

HD	CD	class I		class II		class III		class IV	
		**n**	**%**	**n**	**%**	**n**	**%**	**n**	**%**

PTC	PTC: 30	15	50	14	47	1	3	0	0

	SPTC: 11	4	36	6	55	1	9	0	0

	IFP: 5	0	0	4	80	1	20	0	0

total	46	19		24		3		0	

BN	BN: 17	0	0	5	29	11	65	1	6

	IFP: 19	0	0	8	42	7	37	4	21

total	36			13		18		5	

HD: histological diagnosis. CD: cytological diagnosis. PTC: papillary thyroid carcinoma. SPTC: suspicious for PTC. IFP: indeterminate follicular proliferation. BN: benign nodule. n: number of cases.

- 30 cases cytologically diagnosed as PTC: 15 (50%) were in class I, 14 (47%) in class II, 1 (3%) in class III and none (0%) in class IV.

- 11 cases cytologically diagnosed as SPTC: 4 (36%) in class I, 6 (55%) in class II, 1 (9%) in class III and none (0%) in class 4.

- 5 cases cytologically diagnosed as IFP: 4 (80%) were in class II, 1 (20%) in class III, whereas no case was present in class I and IV.

The cytological diagnoses of the 36 histologically confirmed BN were distributed through the 4 classes as following:

- 17 cases cytologically diagnosed as benign: 5 (29%) were in class II, 11 (65%) in class III, 1 (6%) in class IV, whereas no case was present in class I.

- 19 cases cytologically diagnosed as IFP: 8 (42%) were in class II, 7 (37%) in class III and 4 (21%) in class IV, whereas no case was present in class I.

### BRAF V600E genotyping and c-KIT expression values of cytological samples

The BRAF V600E mutation (Table 2) was found in 54% of the 46 malignant samples (25/46). 48% BRAF V600E mutated samples were in c-KIT class I (12/25), as shown in Table 5. Class II contained the residual 52% of BRAF mutated cases (13/25). Class III and IV had no BRAF mutated cases. BRAF V600E was significantly more present in class I and II (p = 0.00026). No benign samples were BRAF mutated.

**Table 5:** BRAF mutational status according to c-KIT expression classes and to morphological diagnosis

HD	CD	class I		class II		class III		class IV	
		**V600E**	**WT**	**V600E**	**WT**	**V600E**	**WT**	**V600E**	**WT**
		
PTC: 46	PTC: 30	**12**	3	**9**	5	0	1	0	0
	
	SPTC: 11	0	4	**4**	2	0	1	0	0
	
	IFP: 5	0	0	0	4	0	1	0	0

BN: 36	BN: 17	0	0	0	5	0	11	0	1
	
	IFP: 19	0	0	0	8	0	7	0	4

HD: histological diagnosis. CD: cytological diagnosis. WT: wild type. PTC: papillary thyroid carcinoma. SPTC: suspicious for PTC. IFP: indeterminate follicular proliferation.

### c-KIT/BRAF combined molecular analysis in thyroid nodule FNAC

Table 5 shows that four cases of SPTC are in c-KIT class I, whereas four more cases harbor a BRAF V600E mutation. All these 8 cases can be reasonably considered as PTC. At the same time, the four cases with a cytological diagnosis of IFP that are in c-KIT class IV can reasonably considered as benign nodules.

### Role of molecular diagnosis in increasing the diagnostic accuracy of FNAC

As shown in Table 6, if the 8 cases of SPTC in c-KIT class I or hosting a BRAF V600E mutation are moved to the diagnostic group of PTC, the total number of PTC rises from 30 (65%) to 38 (83%), with an advantage in diagnostic accuracy of malignancy of 18%.

**Table 6:** Role of molecular diagnosis in increasing the diagnostic accuracy of FNAC

CD	c-KIT class I	BRAF V600E	c-KIT class IV
SPTC	4	4	

IFP			4

	**CD**	**MD**	**DA**

PTC	30/46: 65%	38/46: 83%	+ 18%

BN	17/36: 47%	21/36: 58%	+ 11%

PTC + BN	47/82: 57%	59/82: 72%	+ 15%

CD: cytological diagnosis. PTC: papillary thyroid carcinoma. SPTC: suspicious for PTC. BN: benign nodule. MD: molecular diagnosis. DA: diagnostic accuracy.

On the other hand, if the 4 cases of IFP in c-KIT class IV are moved to the diagnostic group of BN, the total number of BN rises from 17 (47%) to 21 (58%), with an advantage in diagnostic accuracy of benignity of 11%.

Finally, if we consider both PTC diagnosis and BN diagnosis, the whole diagnostic accuracy gain is of 15% with a statistically significant p-value of 0.03.

## Discussion

Papillary thyroid carcinoma (PTC) is the most common malignancy in thyroid tissue; about 80% of incident thyroid cancers are PTC. Although PTC is usually associated with alterations in the RET/PTC-RAS-BRAF signaling pathway [31,32], the detailed molecular mechanism is unclear. A few papers mentioning a role for c-KIT in thyroid malignancies suggested to perform an analysis of c-KIT expression on thyroid cells obtained by FNAC from benign and malignant thyroid nodules, with the double aim to study a human model of thyroid cancer and, at the same time, to verify if c-KIT expression analysis could be of any clinical interest. The present study evaluated c-KIT expression in a group of thyroid FNAC, assessed later on as malignant or benign by post-surgical histological analysis, and describes the silencing of c-KIT in PTC. These data are in full accordance with a previous paper of Mazzanti *et al*. [18], who were able to identify two classifier models, of 10 and 6 genes respectively, discriminative of PTC and BN, in both of which c-KIT gene was included. In this analysis c-KIT resulted heterogeneously expressed in goiters, whereas PTC were negative. These data give also strength to the report that multiple miRNAs predicted to target c-KIT (miR-221, miR-222, miR-146) are up-regulated in PTC [21,22].

The biological significance of loss of c-KIT in thyroid malignancies is not clear. SCF, the c-KIT ligand, is not mitogenic in primary cultures of thyrocytes even in conjunction with thyroid-stimulating hormone [33], a result which would indicate that SCF/c-KIT pathway may control some aspects of the thyrocyte differentiated phenotype rather than cell division. This would agree with the apparently strong selection for loss of c-KIT expression in neoplastic transformation of thyroid epithelium. This negative selection is in stark contrast with the gain of function due to genetic alterations of tyrosine kinase receptors (TRKs) in other types of cancers, suggesting that TRK signaling pathways may have opposite biological effects in different cell types.

To explore the diagnostic utility of c-KIT expression in thyroid nodules, its expression values were divided in four arbitrarily defined classes, with class I characterized by the complete silencing of the gene. Class I and IV represented the two most informative groups, with 100% of the samples found malignant or benign respectively. Class III was also very informative including 86% of benign samples and having over all the highest statistical significance. On the other hand, in class II the samples belonging to the malignant group were 66%, which resulted non significant. A ROC analysis was performed to measure the diagnostic performance of the model (Figure 2), showing its good efficacy in predicting the malignant events (AUC = 0.9 C.I 95% 0.79-.94; p = 0.001). However, we did not test c-KIT model on unknown samples, since cytological smears employed in this study were obtained from nodules whose histological diagnosis was known. Thus, additional blind samples are required to strengthen the utility of c-KIT test in the preoperative diagnosis.

Moreover, quite interestingly, BRAF V600E mutation, which is a well-known marker for PTC, was found to be statistically more present in the c-KIT classes I and II, while class III and IV did not contain any sample, supporting therefore the association of low c-KIT expression levels to a malignant status.

Molecular Pathology is the modern version of Pathology, where the whole of morphology and molecular alterations represents a powerful approach to diagnosis. In this line, this study aimed to verify the diagnostic potential of c-KIT expression analysis and demonstrated that the combined BRAF mutation and c-KIT expression approach is able to increase the diagnostic accuracy of FNAC of thyroid nodules of 18% for a diagnosis of malignancy and 11% for a diagnosis of benignity.

Despite several carcinomas showed activating mutations of c-KIT gene (GISTs, melanomas, haematopoietic and lymphoid tumors), they have not been described in thyroid tumors and this study revealed a wild type sequence of c-KIT gene in exons 9, 11, 13, and 17.

Finally, as previously published by Jin *et al*. [34], the present paper shows that not only DNA but RNA too can be easily extracted from stained smears of FNAC and easily analyzed by qPCR. c-KIT receptor expression was detectable regardless of the time of specimen collection from the archived material, we were able to successfully use slides prepared 7 years ago and kept in our archives. Moreover all of the smears were independently reviewed by a senior cytopathologist before assessing c-KIT expression, to assure adequate thyroid cell representation of the slides in which c-KIT receptor expression was investigated. The simple method named manual macrodissection and described elsewhere allows to perform molecular analysis only on selected cell population to be studied. This specialized test that may have increased utility as testing based on RNA is becoming more widespread and easily available.

## Conclusion

In summary, we have demonstrated that c-KIT expression-based classification of thyroid lesions is highly accurate and may provide a tool to overcome the difficulties in today's preoperative diagnosis of thyroid suspicious malignancies. We hoped that the test proposed in this paper will be a useful adjunct to the preoperative diagnosis of thyroid nodules.

## Competing interests

The authors declare that they have no competing interests.

## Authors' contributions

ST carried out the study, analyzed the data and wrote the manuscript draft. CM and GB conceived of the manuscript, participated in its design, coordination, analysis and interpretation of data and supervised the writing of the manuscript. PA participated in the statistical data analysis. All the authors made intellectual contributions and approved the final manuscript.

## References

[B1] Hodgson NC Button J Solorzano CC Thyroid cancer: is the incidence still increasing? Ann Surg Oncol 2004 11 1093 1097 10.1245/ASO.2004.03.066 15576834

[B2] Miccoli P Minuto MN Galleri D D'Agostino J Basolo F Antonangeli L Aghini-Lombardi F Berti P Incidental thyroid carcinoma in a large series of consecutive patients operated on for benign thyroid disease ANZ J Surg 2006 76 123 126 10.1111/j.1445-2197.2006.03667.x 16626346

[B3] Udelsman R Chen H The current management of thyroid cancer Adv Surg 1999 33 1 27 10572560

[B4] Mazzaferri EL Management of a solitary thyroid nodule N Engl J Med 1993 328 553 559 10.1056/NEJM199302253280807 8426623

[B5] Hegedus L Clinical practice. The thyroid nodule N Engl J Med 2004 351 1764 1771 10.1056/NEJMcp031436 15496625

[B6] Nikiforov YE Thyroid carcinoma: molecular pathways and therapeutic targets Mod Pathol 2008 21 Suppl 2 S37 43 18437172 10.1038/modpathol.2008.10 PMC2673022

[B7] de Silva CM Reid R Gastrointestinal stromal tumors (GIST): C-kit mutations, CD117 expression, differential diagnosis and targeted cancer therapy with Imatinib Pathol Oncol Res 2003 9 13 19 10.1007/BF03033708 12704441

[B8] Ronnstrand L Signal transduction via the stem cell factor receptor/c-Kit Cell Mol Life Sci 2004 61 2535 2548 10.1007/s00018-004-4189-6 15526160 PMC11924424

[B9] D'Amato G Steinert DM McAuliffe JC Trent JC Update on the biology and therapy of gastrointestinal stromal tumors Cancer Control 2005 12 44 56 15668652 10.1177/107327480501200106

[B10] Miettinen M Makhlouf H Sobin LH Lasota J Gastrointestinal stromal tumors of the jejunum and ileum: a clinicopathologic, immunohistochemical, and molecular genetic study of 906 cases before imatinib with long-term follow-up Am J Surg Pathol 2006 30 477 489 10.1097/00000478-200604000-00008 16625094

[B11] McIntyre A Summersgill B Grygalewicz B Gillis AJ Stoop J van Gurp RJ Dennis N Fisher C Huddart R Cooper C Clark J Oosterhuis JW Looijenga LH Shipley J Amplification and overexpression of the KIT gene is associated with progression in the seminoma subtype of testicular germ cell tumors of adolescents and adults Cancer Res 2005 65 8085 8089 10.1158/0008-5472.CAN-05-0471 16166280

[B12] All-Ericsson C Girnita L Muller-Brunotte A Brodin B Seregard S Ostman A Larsson O c-Kit-dependent growth of uveal melanoma cells: a potential therapeutic target? Invest Ophthalmol Vis Sci 2004 45 2075 2082 10.1167/iovs.03-1196 15223779

[B13] Huang S Luca M Gutman M McConkey DJ Langley KE Lyman SD Bar-Eli M Enforced c-KIT expression renders highly metastatic human melanoma cells susceptible to stem cell factor-induced apoptosis and inhibits their tumorigenic and metastatic potential Oncogene 1996 13 2339 2347 8957075

[B14] Ko CD Kim JS Ko BG Son BH Kang HJ Yoon HS Cho EY Gong G Ahn SH The meaning of the c-kit proto-oncogene product in malignant transformation in human mammary epithelium Clin Exp Metastasis 2003 20 593 597 10.1023/A:1027323210736 14669790

[B15] Tsutsui S Yasuda K Suzuki K Takeuchi H Nishizaki T Higashi H Era S A loss of c-kit expression is associated with an advanced stage and poor prognosis in breast cancer Br J Cancer 2006 94 1874 1878 10.1038/sj.bjc.6603183 16721362 PMC2361342

[B16] Ulivi P Zoli W Medri L Amadori D Saragoni L Barbanti F Calistri D Silvestrini R c-kit and SCF expression in normal and tumor breast tissue Breast Cancer Res Treat 2004 83 33 42 10.1023/B:BREA.0000010694.35023.9e 14997053

[B17] Natali PG Berlingieri MT Nicotra MR Fusco A Santoro E Bigotti A Vecchio G Transformation of thyroid epithelium is associated with loss of c-kit receptor Cancer Res 1995 55 1787 1791 7536131

[B18] Mazzanti C Zeiger MA Costouros NG Umbricht C Westra WH Smith D Somervell H Bevilacqua G Alexander HR Libutti SK Using gene expression profiling to differentiate benign versus malignant thyroid tumors Cancer Res 2004 64 2898 2903 10.1158/0008-5472.CAN-03-3811 15087409

[B19] Prasad NB Somervell H Tufano RP Dackiw AP Marohn MR Califano JA Wang Y Westra WH Clark DP Umbricht CB Libutti SK Zeiger MA Identification of genes differentially expressed in benign versus malignant thyroid tumors Clin Cancer Res 2008 14 3327 3337 10.1158/1078-0432.CCR-07-4495 18519760 PMC3086681

[B20] Rosen J He M Umbricht C Alexander HR Dackiw AP Zeiger MA Libutti SK A six-gene model for differentiating benign from malignant thyroid tumors on the basis of gene expression Surgery 2005 138 1050 1056 discussion 1056-1057 10.1016/j.surg.2005.09.010 16360390

[B21] He H Jazdzewski K Li W Liyanarachchi S Nagy R Volinia S Calin GA Liu CG Franssila K Suster S Kloos RT Croce CM de la Chapelle A The role of microRNA genes in papillary thyroid carcinoma Proc Natl Acad Sci USA 2005 102 19075 19080 10.1073/pnas.0509603102 16365291 PMC1323209

[B22] Mazeh H Mizrahi I Halle D Ilyayev N Stojadinovic A Trink B Mitrani-Rosenbaum S Roistacher M Ariel I Eid A Freund HR Nissan A Development of a microRNA-based molecular assay for the detection of papillary thyroid carcinoma in aspiration biopsy samples Thyroid 2011 21 111 118 10.1089/thy.2010.0356 21275764

[B23] Hamberger B Gharib H Melton LJ Goellner JR Zinsmeister AR Fine-needle aspiration biopsy of thyroid nodules. Impact on thyroid practice and cost of care Am J Med 1982 73 381 384 10.1016/0002-9343(82)90731-8 7124765

[B24] Suen KC How does one separate cellular follicular lesions of the thyroid by fine-needle aspiration biopsy? Diagn Cytopathol 1988 4 78 81 10.1002/dc.2840040119 3378490

[B25] Caraway NP Sneige N Samaan NA Diagnostic pitfalls in thyroid fine-needle aspiration: a review of 394 cases Diagn Cytopathol 1993 9 345 350 10.1002/dc.2840090320 8519202

[B26] Goellner JR Problems and pitfalls in thyroid cytology Monogr Pathol 1997 75 93 9249821

[B27] Goellner JR Gharib H Grant CS Johnson DA Fine needle aspiration cytology of the thyroid, 1980 to 1986 Acta Cytol 1987 31 587 590 3673463

[B28] Ravetto C Colombo L Dottorini ME Usefulness of fine-needle aspiration in the diagnosis of thyroid carcinoma: a retrospective study in 37,895 patients Cancer 2000 90 357 363 10.1002/1097-0142(20001225)90:6<357::AID-CNCR6>3.0.CO;2-4 11156519

[B29] Marchetti I Lessi F Mazzanti CM Bertacca G Elisei R Coscio GD Pinchera A Bevilacqua G A morpho-molecular diagnosis of papillary thyroid carcinoma: BRAF V600E detection as an important tool in preoperative evaluation of fine-needle aspirates Thyroid 2009 19 837 842 10.1089/thy.2009.0074 19534623

[B30] Xing M BRAF mutation in papillary thyroid cancer: pathogenic role, molecular bases, and clinical implications Endocr Rev 2007 28 742 762 10.1210/er.2007-0007 17940185

[B31] Kimura ET Nikiforova MN Zhu Z Knauf JA Nikiforov YE Fagin JA High prevalence of BRAF mutations in thyroid cancer: genetic evidence for constitutive activation of the RET/PTC-RAS-BRAF signaling pathway in papillary thyroid carcinoma Cancer Res 2003 63 1454 1457 12670889

[B32] Melillo RM Castellone MD Guarino V De Falco V Cirafici AM Salvatore G Caiazzo F Basolo F Giannini R Kruhoffer M Orntoft T Fusco A Santoro M The RET/PTC-RAS-BRAF linear signaling cascade mediates the motile and mitogenic phenotype of thyroid cancer cells J Clin Invest 2005 115 1068 1081 15761501 10.1172/JCI22758 PMC1062891

[B33] Zsebo KM Williams DA Geissler EN Broudy VC Martin FH Atkins HL Hsu RY Birkett NC Okino KH Murdock DC Stem cell factor is encoded at the Sl locus of the mouse and is the ligand for the c-kit tyrosine kinase receptor Cell 1990 63 213 224 10.1016/0092-8674(90)90302-U 1698556

[B34] Jin L Lloyd RV Nassar A Lappinga PJ Sebo TJ Swartz K Seys AR Erickson-Johnson MR Roth CW Evers BR Oliveira AM Zhang J HMGA2 expression analysis in cytological and paraffin-embedded tissue specimens of thyroid tumors by relative quantitative RT-PCR Diagn Mol Pathol 2011 20 71 80 10.1097/PDM.0b013e3181ed784d 21532495

